# Assessing the Mobility Disruption of *Tribolium confusum* Under Sublethal Concentrations of Chlorfenapyr Through Video Tracking Software

**DOI:** 10.3390/insects17080801

**Published:** 2026-08-03

**Authors:** Constantin S. Filintas, Nickolas G. Kavallieratos, Maria C. Boukouvala

**Affiliations:** Laboratory of Agricultural Zoology and Entomology, Agricultural University of Athens, 75 Iera Odos str., 11855 Athens, Greece; kfilintas@aua.gr (C.S.F.); nick_kaval@aua.gr (N.G.K.)

**Keywords:** pyrrole, stored-product pest, Tenebrionidae, automated system, behavior

## Abstract

*Tribolium confusum* is a destructive global pest of processed grains, causing severe economic damages in storages. After the application of synthetic insecticides to surfaces, they degrade, allowing insects to come in contact with lower concentrations of the toxicant. This study focused on the investigation of the sublethal impacts of the pyrrole insecticide chlorfenapyr across different surface types (plastic, glass, metal, cement) on *T. confusum*. Using Toxtrac for automated video tracking, we quantified locomotor traits of *T. confusum* adult males and females. Chlorfenapyr surface treatment at LC_10_ and LC_30_ suppressed the locomotor activity of *T. confusum* adults. Important mobility traits were quantified using ToxTrac analysis, such as average speed, average acceleration, total distance, exploration rate, mobility average speed, and total time frozen. In the untreated groups, beetles displayed high levels of activity, with average speeds exceeding 50 mm/s on glass, plastic, and cement. After insecticide exposure, all locomotor traits declined across all surface types, except for total time frozen, that showed a positive correlation with the increase in concentration of chlorfenapyr. Treatments on non-porous materials (plastic, glass, metal) caused higher mobility suppression in both sexes in comparison to respective treatments on cement. These effects were more prevalent at LC_30_ vs. LC_10_. Chlorfenapyr induced a strong, negative effect on the mobility behavior of *T. confusum* adults across all tested surfaces, highlighting the value of automated behavioral tracking for detecting and quantifying key mobility impairment on specific traits relevant to stored-product pest management.

## Simple Summary

This study investigated the effects of chlorfenapyr at LC_10_ and LC_30_ on the locomotor activity of males and females of *Tribolium confusum* on plastic, glass, metal, and cement surfaces. Mobility was recorded in videos that were analyzed by ToxTrac software. The evaluated behavioral traits included average speed, average acceleration, total distance traveled, and total time frozen. Chlorfenapyr exposure at both concentrations consistently affected locomotor activity in both sexes, across all surfaces. Plastic, glass, and metal surfaces procured significant mobility suppression, with pronounced effects at LC_30_. These findings highlight the importance and reliability of automated tracking as a sensitive and informative tool for evaluating the behavioral alterations of *T. confusum* under insecticide stress.

## 1. Introduction

The tenebrionid *Tribolium confusum* Jacquelin du Val (Coleoptera) is a major secondary pest of stored products, causing considerable financial losses in food processing centers, mills, and commercial storage facilities [1,2]. The direct damage caused by *T. confusum* is a result of feeding habits on finely ground or broken starch materials [3]. This noxious species has attained a worldwide distribution and is well adapted to cooler regions than one other tenebrionid flour beetle, *Tribolium castaneum* (Herbst) (Coleoptera) [4]. As a secondary storage pest, it lacks the ability to penetrate the hard seed coat of whole grains, but once the grain is processed down, it can infest a wide array of products, including flours, cereals, spices, and chocolate products [5,6]. This feeding activity leads to a direct loss of product mass and quality. However, the indirect damage is often far more significant and complex. Infestations lead to the presence of all life stages, such as eggs, larvae, pupae, and adults, causing food contamination with their bodies, feces, and secretions [7]. *Tribolium confusum* is known for secreting benzoquinones from a pair of abdominal glands, producing a pungent odor, which can cause discoloration to the grain, hindering its quality and consumer appeal [8].

The use of commercial synthetic insecticides has traditionally been the cornerstone of pest management strategies in stored-product protection. Compounds such as organophosphates, pyrethroids, and fumigants have been widely applied due to their broad-spectrum activity and relatively fast insecticidal effects [9,10]. However, the excessive and prolonged use of these chemicals has raised several concerns, including the development of insect resistance, the presence of chemical residues in food products, and negative consequences on both human health and the environment [11,12]. These challenges have prompted researchers and the pest management industry to search for compounds with novel modes of action. One such compound is chlorfenapyr, a pyrrole derivative that functions as a pro-insecticide, i.e., becoming toxic only after metabolic activation within the insect [13]. Its unique mode of action is based on the disruption of mitochondrial oxidative phosphorylation and thereby halting energy production [14,15]. This contact insecticide has been extensively studied for toxicity against major stored-product pests, including *Tribolium* spp. [16,17,18]. The effectiveness of contact insecticides inside storage facilities is influenced by several key factors, primarily the type of treated surface, quantity of insecticide formulation, and environmental conditions, such as temperature and humidity [19,20]. Specifically, surface characteristics, such as porosity and texture can heavily affect the availability of the insecticide and thus facilitate or hinder its contact with the target insect [21,22].

Commercial contact insecticides applied for insect pest control usually degrade in storage settings after persisting for some time, resulting in concentrations lower than the commercially suggested for insect pest toxicity [23,24]. Exposure to concentrations below the lethal threshold does not necessarily result in mortality but it (a) reduces fitness, walking speed, climbing, flying, reproduction, foraging or feeding; (b) induces disorientation or irregular movement trajectories; (c) prevents them from finding suitable oviposition sites or dispersing; and (d) suppresses population growth over time [25,26,27,28,29,30,31,32]. Conversely, sublethal concentrations may temporarily stimulate hyperactivity and increase energy expenditure [33].

Behavioral assays have gained considerable traction in recent years in ecotoxicological research as tools for capturing variable biological responses [34,35]. They are highly sensitive and fast methods to assess various effects of exogenous stressors on animal organisms, compared to conventional approaches that estimate mortality, reproduction, and development [36]. Concurrently, behavioral studies employing video tracking to quantify key physiological effects and patterns are undergoing rapid development and application in recent years [32,37,38]. ToxTrac is a free online tracking software compatible with a real-time application, requiring no specific knowledge of the geometry of the tracked objects. ToxTrac is able to measure behavioral parameters necessary for calculating locomotor activity, e.g., average speed, acceleration, and distance traveled per time unit [39]. To date, this software has been successfully used to evaluate the mobility characteristics of pests of public importance and field pests [40,41,42]. To our knowledge, there is no data available regarding the mobility of *T. confusum* on different surface materials when treated with insecticides. Therefore, the aim of this study was to investigate, for the first time, six mobility traits of *T. confusum* adult males and females after their exposure to sublethal concentrations of chlorfenapyr on four types of surfaces, implementing ToxTrac as a tracking software.

## 2. Materials and Methods

### 2.1. Insect Rearing

The male and female adults of *T. confusum* that were used in the bioassays were obtained from the AUA (Agricultural University of Athens) and LAZEN (Laboratory of Agricultural Zoology and Entomology). *Tribolium confusum* individuals were reared on insecticide-free wheat flour mixed with brewer’s yeast (5%). The colonies were maintained in controlled conditions (65% relative humidity (RH) and 30 °C) and continuous darkness [43]. Insects were sexed at the pupa stage using the differences in the urogomphi of each sex [44]. Then they were kept separately, by sex, in jars with rearing medium until the start of the bioassays. Adults selected for the bioassays were approximately four weeks old.

### 2.2. Insecticide

The commercial formulation Phantom EC (BASF Hellas, Athens, Greece), containing 21.45% chlorfenapyr as the active ingredient (a.i.), was utilized in the bioassays.

### 2.3. Mortality Assays for LC Determination

Contact toxicity bioassays were conducted to determine the sublethal concentrations of chlorfenapyr against adults of *T. confusum* on four surfaces (plastic, glass, cement, and metal) [32]. Standard plastic and glass Petri dishes (Ø 80 mm × 15 mm) were used for the plastic and glass surfaces. Individual galvanized metal surfaces were cut into circular disks to fit exactly into the base of plastic dishes [45]. Additionally, another batch of plastic dishes was prepared with a layer of cement (Durostick, Aspropyrgos, Greece) covering their bottoms [46]. Each surface was treated separately using an airbrush, applying 1 mL of chlorfenapyr solution at various dilutions, using distilled water (i.e., 0.0025, 0.0013, 0.0008, 0.000625, 0.0005, and 0.00042 mg a.i./cm^2^). Treated surfaces were then left to dry for 24 h at 30 °C. Subsequently, 20 adult *T. confusum* were introduced onto each surface and then maintained in controlled conditions at 30 °C, 65% RH, and continuous darkness (6 concentrations × 5 replications = 30 dishes/surface) [47]. After 24 h of exposure, mortality was recorded for each treatment and surface. The same procedure was carried out on the control group using only distilled water. The whole procedure was repeated three times in total, as independent biological replicates using insects from different cohorts and preparing new solutions and surfaces (6 concentrations × 5 replications × 3 repetitions × 4 surfaces = 360 dishes).

### 2.4. Recording Setup and Mobility Bioassays

**Figure 1:**
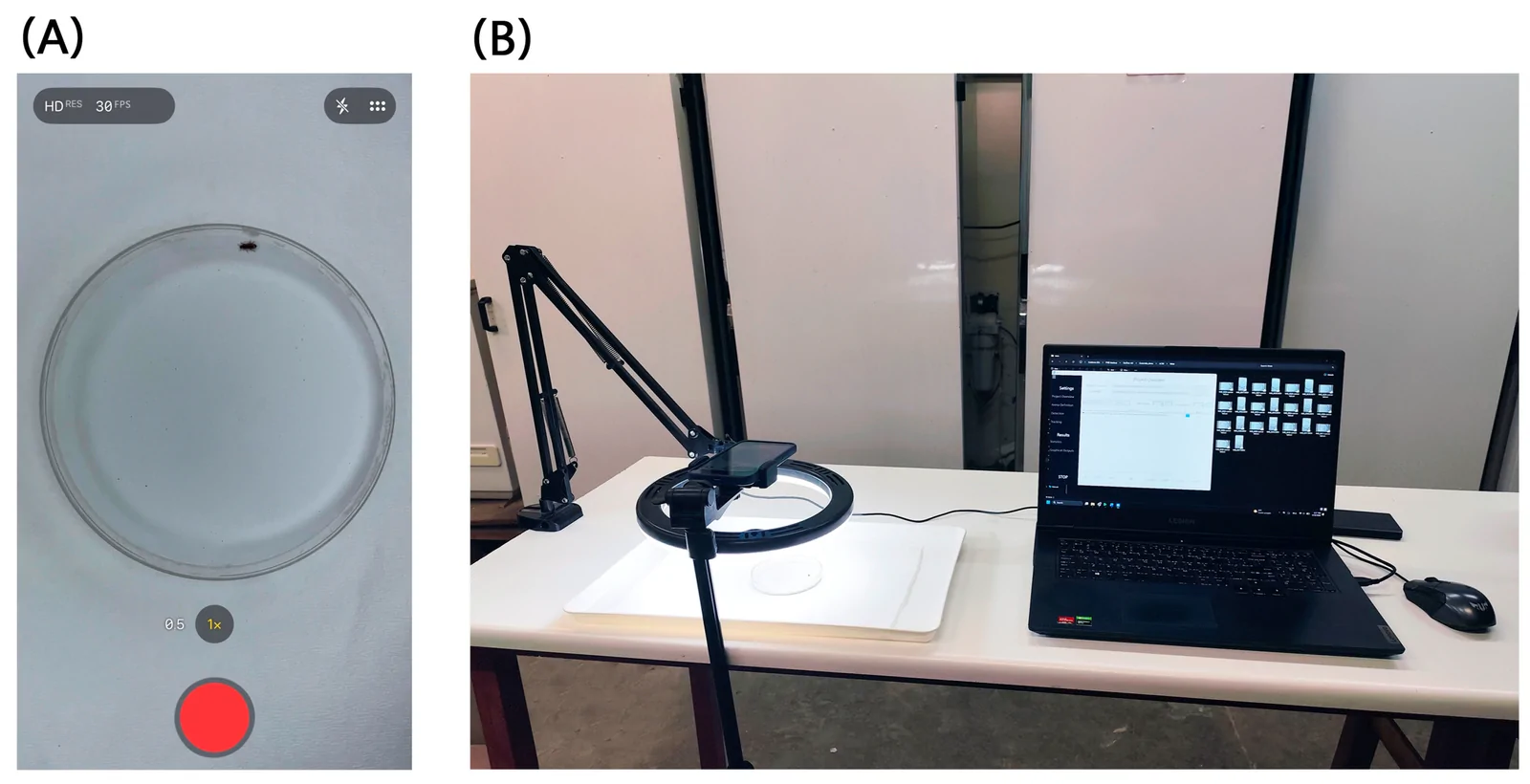
Experimental setup used for recording *Tribolium confusum* mobility assays: (**A**) smartphone camera interface depicting settings and the arena used for video acquisition, (**B**) total recording setup consisting of a smartphone mounted above the arena with a ring light and a laptop used for video processing in ToxTrac.

For the mobility trials, per type of surface, 20 male adults or 20 female adults were exposed for 24 h to the LC_10_ of chlorfenapyr, following the procedure described in the previous section. The same process was followed for LC_30_ and control treated with distilled water, which served as controls [46]. Following exposure, treated individuals were removed from the dishes and kept by sex in untreated dishes of the same surface. Then, a single insect (male or female) of the corresponding surface/concentration was placed in a new untreated arena (Ø 80 mm × 15 mm) made of the respective surface material and left to acclimate for 60 s [32]. The mobility behavior of each individual was then recorded for five min. The process was repeated until 30 videos (i.e., 30 different individuals) were acquired for each surface, sex, and concentration. A total of 720 individuals were recorded (30 individuals × 2 sexes × 3 treatments (including control) × 4 surfaces). The recording setup consisted of a smartphone camera (iPhone 12 camera, HD, 30 fps) (Apple Inc., Cupertino, CA, United States) mounted on a tripod (Ugreen Group Limited, Shenzhen, China) (Figure 1). The phone camera was placed at a height of 14 cm above the arena, accompanied by a ring light to ensure consistent and adequate illumination during video acquisition. The lighting conditions consisted of white light at the brightest setting, measured at 1520 lux (Umarex, Arnsberg, Germany). ToxTrac operates on the basis of a contrasted background (e.g., lighter background, darker tracked object (insect)). Thus, it is able to identify the insect’s trajectory based on the darker pixels per frame. Videos were acquired in constant room settings.

All videos were uploaded into ToxTrac (version 1.4). Then the virtual arena size was defined and validation of the insect detection at a 100% rate was performed manually to ensure tracking. The calibration procedure was the same for all acquired videos. Following ToxTrac’s analysis, mobility datasets from each recorded video were extracted onto Excel spreadsheets. Every dataset had to include 9000 visible frames and a 100% visibility rate in order to be eligible for the subsequent analysis, after standardization in preliminary video recordings. Each dataset was transferred into tables based on surface type. The selected mobility parameters included: (1) total distance: cumulative distance covered during the recording period, (2) average speed: mean velocity of the insect, (3) average acceleration: mean rate at which velocity changes with time, (4) exploration rate: the proportion of the arena explored, (5) mobility average speed: mean velocity during active movement periods, and (6) total frozen time: cumulative duration of complete immobility.

### 2.5. Statistical Analysis

LC_10_, LC_30_, and LC_50_ of chlorfenapyr were determined separately for each surface using probit analysis with 95% confidence intervals (CI) [48] implemented in the statistical software R (version 4.5.0) with the ecotox package (Code S1) [49]. Collected data on the effects of chlorfenapyr on the mobility of adult male and female *T. confusum* were tested for normality using the Shapiro–Wilk test [50] and for homoscedasticity using Levene’s test [51]. Data were then transformed using log(x + 1) to achieve homogeneity of variance and approximate normality [52]. The effects of chlorfenapyr were evaluated using a three-way analysis of variance (three-way ANOVA), with treatment (control, chlorfenapyr LC_10_ and LC_30_), sex (male and female), and surface type (plastic, glass, metal, and cement) as the main effects. All possible interactions among the main effects were included in the model. ANOVA was conducted using the software JMP version 16.2 by selecting “fit model” and “full factorial” [53,54]. The JMP Graph Builder application was used to construct the graphs, which allowed the data to be displayed in boxplot format, highlighting the median, quartiles, and range of values (minimum–maximum). Mean comparisons were performed using Tukey’s HSD test on transformed data, at a significance level of 0.05 [55].

## 3. Results

### 3.1. LC Values of Chlorfenapyr onCement, Plastic, Glass, and Metal Surfaces

According to the contact toxicity bioassays, estimated LC_10_, LC_30_, and LC_50_ values varied among surfaces. The highest values were recorded on cement, i.e., 0.00838, 0.0131, and 0.0178 mg a.i./cm^2^ followed by plastic, i.e., 0.00475, 0.00822, and 0.0120 mg a.i./cm^2^, for LC_10_, LC_30_, and LC_50_, respectively. Lower values were recorded on glass, i.e., 0.00403, 0.00688, and 0.00996 mg a.i./cm^2^ and on metal, i.e., 0.00463, 0.00763, and 0.0108 mg a.i./cm^2^, for LC_10_, LC_30_, and LC_50_, respectively (Table 1).

### 3.2. Mobility Traits of T. confusum Males and Females on Different Surfaces

The main effects surface or treatment and the interactions surface × treatment and sex × surface × treatment were significant for average speed, average acceleration, and total distance of adult *T. confusum* individuals (Table 2). The main effect of sex and the interaction sex × treatment were not significant for the exploration rate of *T. confusum* individuals. The main effect surface and all associated interactions, with the exception of sex × treatment, were significant for the mobility average speed of *T. confusum* individuals. Finally, all main effects and the interactions material × treatment and sex × material × treatment were significant for the total time frozen.

#### 3.2.1. Average Speed

**Figure 2:**
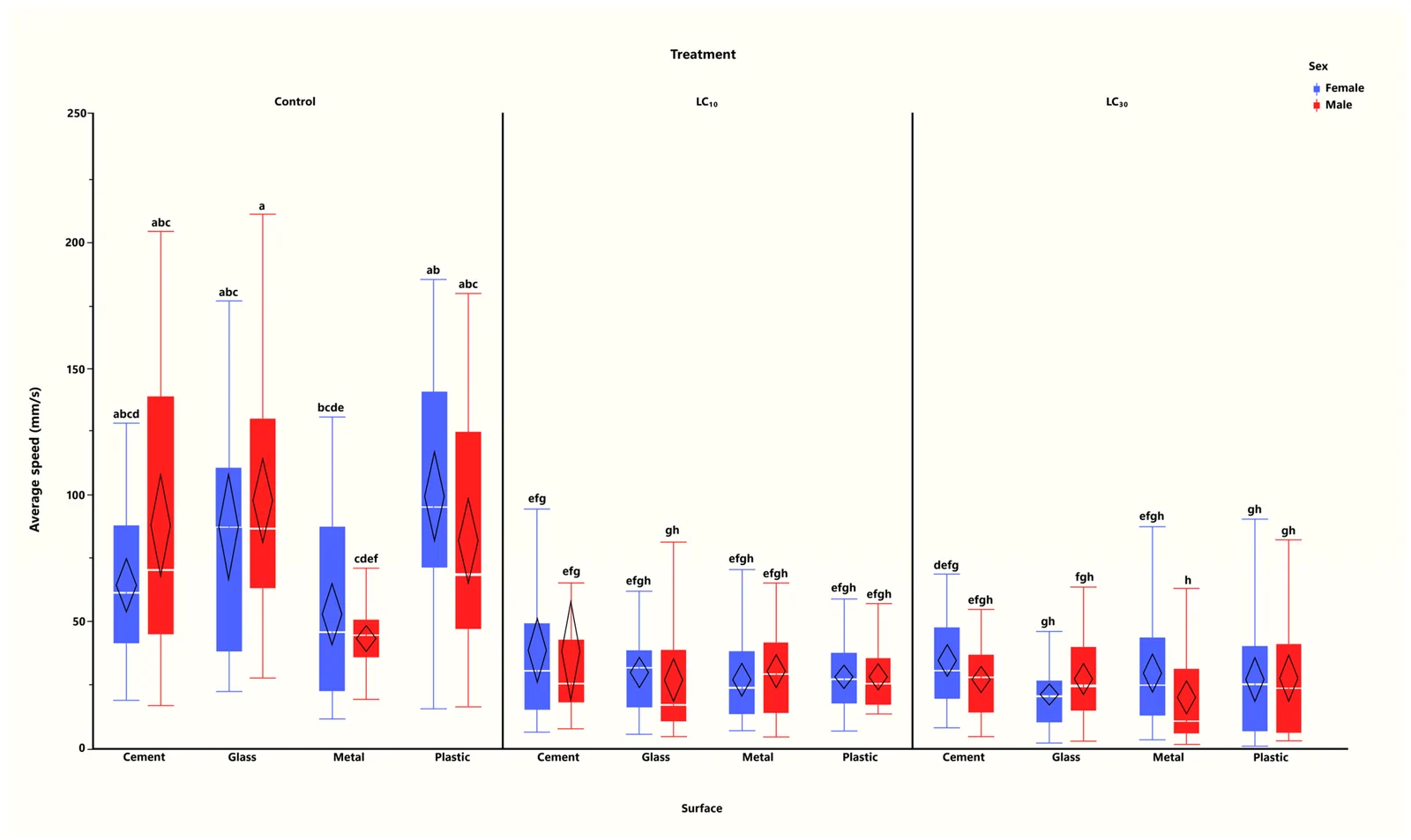
Average speed of male and female adults of *Tribolium confusum* on cement, glass, metal, and plastic surfaces following exposure to chlorfenapyr at LC_10_, LC_30_, or control. Boxes, horizontal lines within boxes, and whiskers above and below boxes indicate the interquartile range, the median values, and the data range, respectively. Per box, each confidence diamond corresponds to the mean value ± SE. Different lowercase letters indicate significant differences among treatments, surfaces, and sexes (*F* = 17.9, df = 23, 719, *p* < 0.01) (sample size *n* = 30).

In the control groups, both sexes demonstrated high levels of activity and variability ranging from 42.66 mm/s in males on metal to 98.65 mm/s in females on plastic. Significantly higher values were consistently observed on plastic, glass and cement for males (>80 mm/s), whereas lower speeds were recorded in females on cement and metal. Following exposure to chlorfenapyr, average speed declined significantly across all tested surfaces and both sexes, compared to the control. At LC_10_, values were reduced to a range of 26.32–37.78 mm/s, while LC_30_ further decreased mobility to 19.53–33.81 mm/s (Table S1). Although both LC_10_ and LC_30_ treatments caused a drastic decrease in average speed in both sexes, differences among surfaces and sexes were not significant (Figure 2).

#### 3.2.2. Average Acceleration

**Figure 3:**
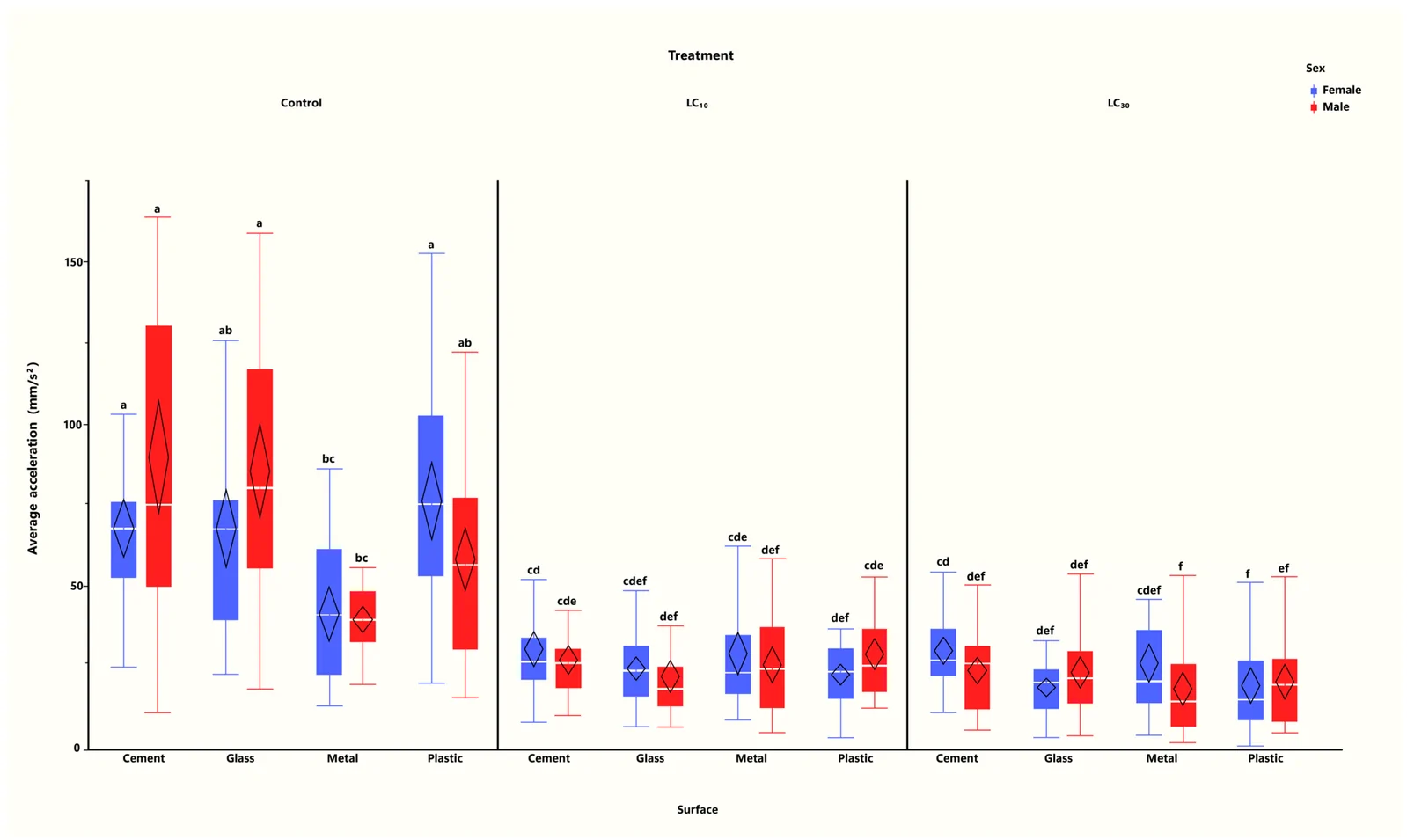
Average acceleration of male and female adults of *Tribolium confusum* on cement, glass, metal, and plastic surfaces following exposure to chlorfenapyr at LC_10_, LC_30_, or control. Boxes, horizontal lines within boxes, and whiskers above and below boxes indicate the interquartile range, the median values, and the data range, respectively. Per box, each confidence diamond corresponds to the mean value ± SE. Different lowercase letters indicate significant differences among treatments, surfaces, and sexes (*F* = 27.8, df = 23, 719, *p* < 0.01) (sample size *n* = 30).

In the control groups, males and females exhibited high average acceleration on cement (89.8 mm/s^2^ and 68.4 mm/s^2^, respectively) and glass (68.2 mm/s^2^ and 63.9 mm/s^2^, respectively) and plastic surfaces (87.9 mm/s^2^ and 63.8 mm/s^2^, respectively) (Table S1, Figure 3). However, on metal, the mean recorded average acceleration was significantly lower for both males and females (39.6 mm/s^2^ and 41.9 mm/s^2^, respectively). Chlorfenapyr significantly reduced the average acceleration in both sexes, only for plastic, glass and cement for both treatments. Both LC treatments had a similar uniform effect on average acceleration, reducing it to levels ranging between 18.3 and 31 mm/s^2^ across surfaces. Differences between LC_10_ and LC_30_ on all surfaces were subtle and non-significant (Figure 3).

#### 3.2.3. Total Distance

**Figure 4:**
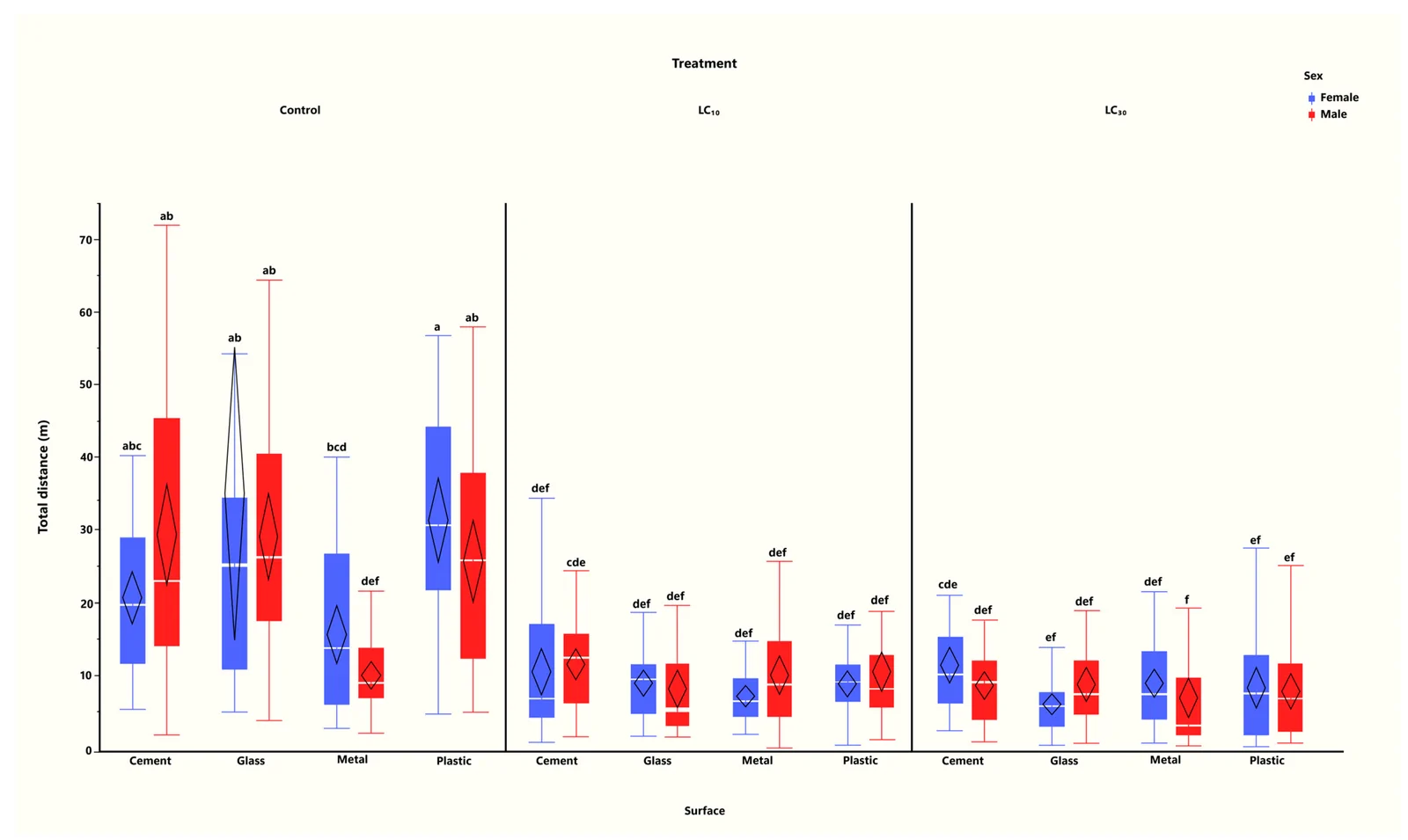
Total distance traveled by male and female adults of *Tribolium confusum* on cement, glass, metal, and plastic surfaces following exposure to chlorfenapyr at LC_10_, LC_30_, or control. Boxes, horizontal lines within boxes, and whiskers above and below boxes indicate the interquartile range, the median values, and the data range, respectively. Per box, each confidence diamond corresponds to the mean value ± SE. Different lowercase letters indicate significant differences among treatments, surfaces, and sexes (*F* = 15.9, df = 23, 719, *p* < 0.01) (sample size *n* = 30).

For both sexes in controls, total distance was significantly lower on metal than glass, plastic and cement (Figure 4). Females on glass in the control group recorded an average total distance of 35.10 m, whereas the corresponding LC_10_ and LC_30_ values were significantly reduced to 9.01 m and 6.01 m, respectively. Similar significant reductions were observed for males on the same surface, i.e., 29.0 m in control vs. 8.2 m at LC_10_ and 8.7 m at LC_30_, respectively. On cement, females and males exhibited significant reductions from control values of 20.6 and 29.0 to 11.3 m and 8.6 m, respectively (Table S1). No significant differences were detected between LC_10_ or LC_30_ and among exposed combinations across surfaces or sexes. Overall, significant differences were recorded among controls and exposed insects of both sexes to both LC treatments (Figure 4).

#### 3.2.4. Exploration Rate

**Figure 5:**
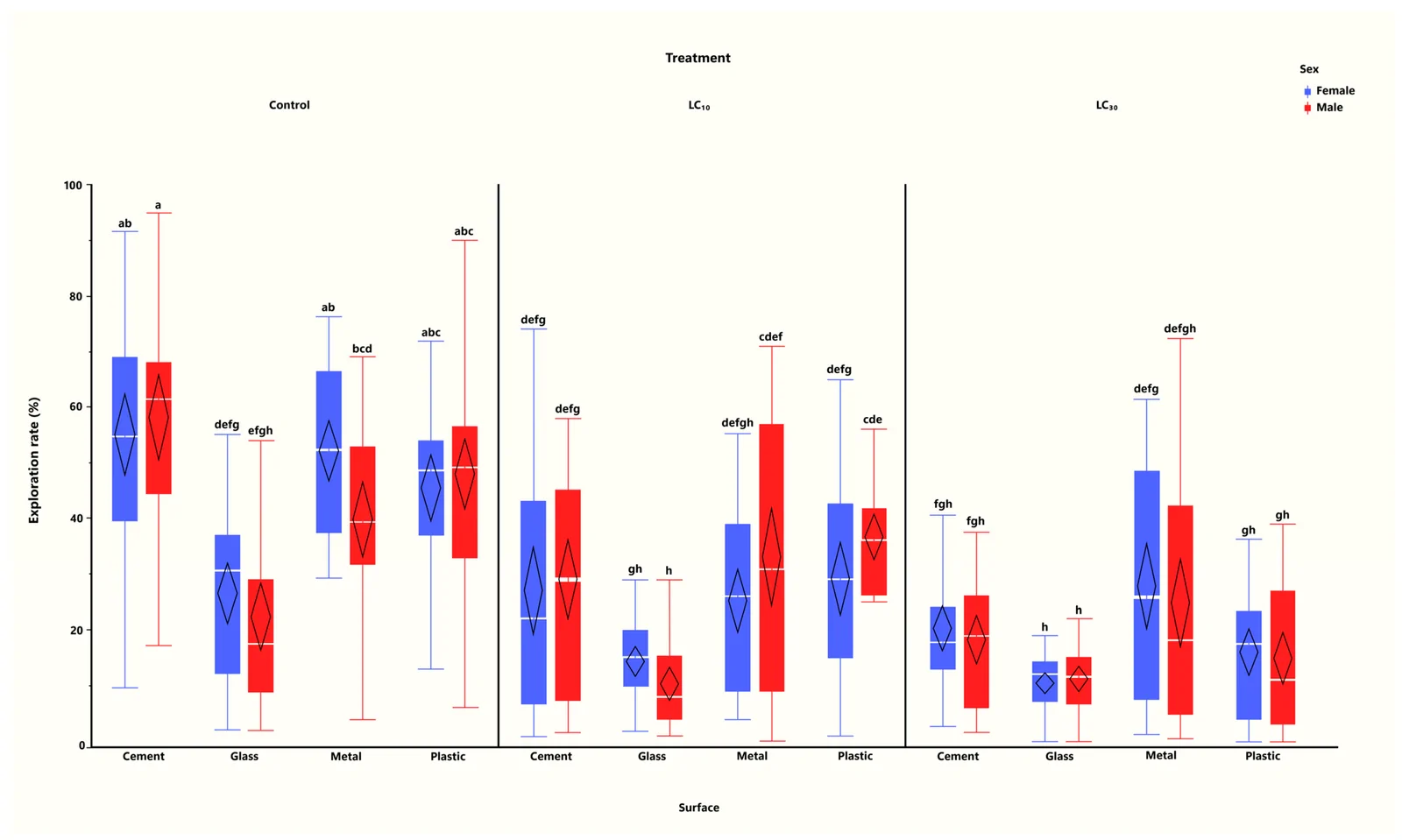
Exploration rate of male and female adults of *Tribolium confusum* on cement, glass, metal, and plastic surfaces following exposure to chlorfenapyr at LC_10_, LC_30_, or control. Boxes, horizontal lines within boxes, and whiskers above and below boxes indicate the interquartile range, the median values, and the data range, respectively. Per box, each confidence diamond corresponds to the mean value ± SE. Different lowercase letters indicate significant differences among treatments, surfaces, and sexes (*F* = 24.0, df = 23, 719, *p* < 0.01) (sample size *n* = 30).

In the control groups, the exploration rate on glass was significantly lower than on cement for both sexes (Figure 5). Both metal and plastic exploration rate values were higher than glass without significant differences. Following chlorfenapyr exposure at LC_10_ and LC_30_, exploration rates were significantly reduced only on cement for males (18.1 and 28.8%, respectively) and females (27.1 and 20.2%, respectively), when compared to controls (Table S1). At LC_10_, on glass, there were no significant differences in exploration rate, while only females exhibited a significant reduction at LC_30_ compared to the control (Figure 5).

#### 3.2.5. Mobility Average Speed

**Figure 6:**
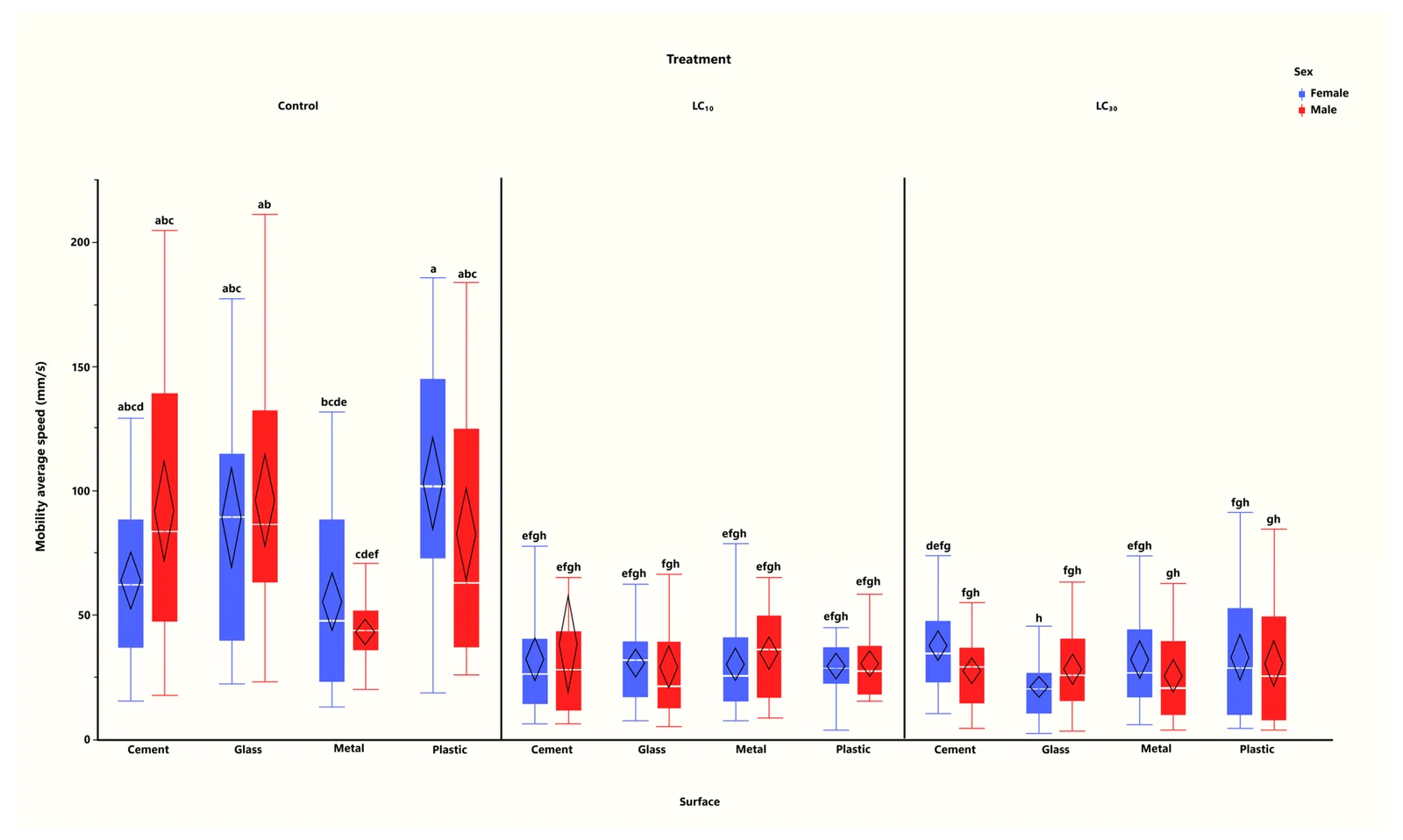
Mobility average speed of male and female adults of *Tribolium confusum* on cement, glass, metal, and plastic surfaces following exposure to chlorfenapyr at LC_10_, LC_30_, or control. Boxes, horizontal lines within boxes, and whiskers above and below boxes indicate the interquartile range, the median values, and the data range, respectively. Per box, each confidence diamond corresponds to the mean value ± SE. Different lowercase letters indicate significant differences among treatments, surfaces, and sexes (*F* = 16.9, df = 23, 719, *p* < 0.01) (sample size *n* = 30).

In control groups, there were no significant differences among cement, glass and plastic for both sexes (Figure 6). Generally, males and females on metal exhibited reduced mobility and average speed, compared to other surfaces in the controls. Both LC treatments in both sexes resulted in significant reductions in plastic (females: 29.1 mm/s for LC_10_ and 32.9 mm/s for LC_30_; males: 30.4 mm/s for LC_10_ and 30.3 mm/s for LC_30_) and glass (females: 30.4 mm/s for LC_10_ and 20.8 mm/s for LC_30_; males: 28.9 mm/s for LC_10_ and 28.1 mm/s for LC_30_) vs. controls (Table S1). Differences between LC_10_ and LC_30_ across all surfaces and sexes were not significant (Figure 6).

#### 3.2.6. Total Time Frozen

**Figure 7:**
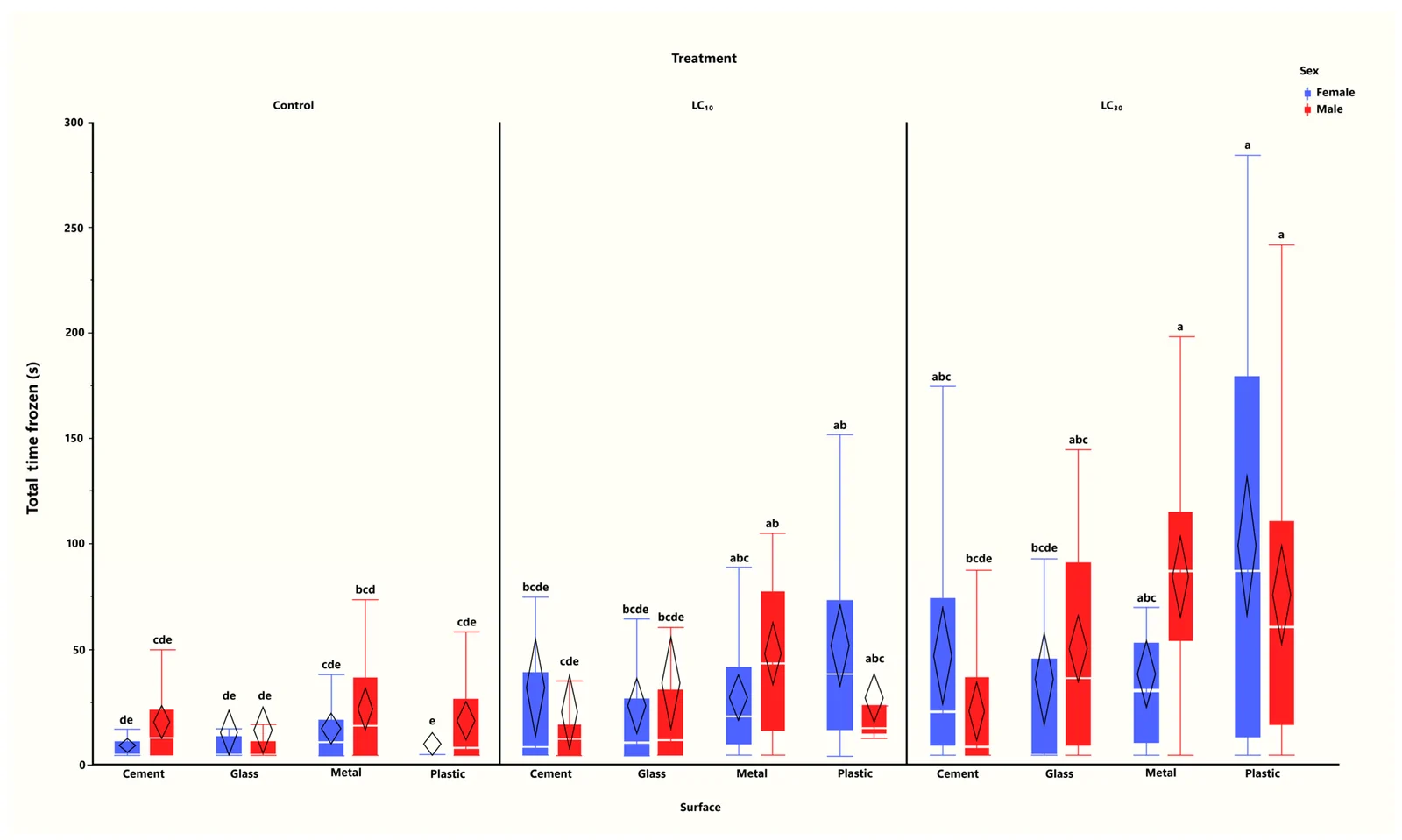
Total time frozen of male and female adults of *Tribolium confusum* on cement, glass, metal, and plastic surfaces following exposure to chlorfenapyr at LC_10_, LC_30_, or control. Boxes, horizontal lines within boxes, and whiskers above and below boxes indicate the interquartile range, the median values, and the data range, respectively. Per box, each confidence diamond corresponds to the mean value ± SE. Different lowercase letters indicate significant differences among treatments, surfaces, and sexes (*F* = 10.6, df = 23, 719, *p* < 0.01).

Freezing duration remained consistently low regardless of surface type and sex, in the controls (Figure 7). Treated insects at LC_10_ and LC_30_ resulted in increased total time frozen of both sexes on all types of surfaces. The highest total freezing time under LC_30_ was observed in females on plastic (100.9 s) while the lowest was in males on cement (21.5 s) (Table S1). No significant differences were detected between LC_10_ and LC_30_ within any surface or sex (Figure 7).

## 4. Discussion

It is well known that exogenous stimuli affect biological traits in invertebrates [56]. Recent advances in behavioral toxicology have demonstrated that behavioral effects induced by various exogenous stimuli, such as insecticidal agents, can be successfully assessed through computerized analysis systems [57,58,59]. Thus, the transition to more automated approaches allows for customizable recording periods and the precise quantification of behavioral traits, producing highly reliable and reproducible behavioral data for subsequent analyses that would have been really difficult and laborious to assess through manual observation alone [60,61]. For example, Ratnayake et al. [62] demonstrated that a motion-based compression and tracking system achieved a high detection rate (97.5%). An elevated detection rate is vital because it ensures that most recorded behavioral events are correctly captured and tracked, minimizing data loss. Automated systems also capture rapid and subtle movement disruptions that are often missed during conventional observation approaches. It is also possible to distinguish different behavioral components within the same recording; that is especially important in toxicological studies where sublethal effects of insecticides often appear as brief and repeated interruptions of movement [32]. Therefore, in this study, the use of ToxTrac as a motion analysis software offered an objective and reliable approach for evaluating certain parameters of the locomotory activity of *T. confusum* under chlorfenapyr stress. By combining standardized exposure conditions with recording in a uniform arena, documented differences can reliably be attributed to treatment effects. It provides continuous, trajectory-based tracking, which allows mobility behavior to be quantified in a detailed way rather than relying on occasional visual estimates [39]. Recent behavioral studies conducted on stored-product insect species implementing the tracking software ToxTrac highlighted its versatile nature when assessing mobility under different reliable scenarios. Specifically, Scharf et al. [63] found that arena characteristics affected *T. castaneum* movement, with alterations in the covered distance, directionality, and the tendency to stay near corners (thigmotaxis). Furthermore, analysis of data obtained from walking assays, evaluating the movement behavior of *Sitophilus zeamais* Motschulsky (Coleoptera: Curculionidae) after exposure to fumonisins of *Fusarium verticillioides*, revealed a repellent effect on the exposed insects [64].

According to our findings, toxicity in mortality assays for LC estimation differed clearly among the tested surface materials, indicating that surface properties play an important role in the effectiveness of chlorfenapyr. The lowest efficacy was observed on cement, where higher concentrations were required to achieve comparable levels of mortality. This is likely related to the high porosity of cement, which can absorb part of the applied insecticide solution and reduce its availability to target insects [65]. In contrast, the treatments were more effective for the non-porous tested surfaces (i.e., glass, metal, and plastic), where lower concentrations were required to induce mortality, because they likely allowed greater contact between the insects and the a.i., enhancing the uptake [19]. Although the differences among the non-porous materials were small, glass appeared to be the most effective surface, considering insecticidal performance.

In this study, a notable outcome from the behavioral trials was the consistency of mobility suppression across both LC treatments, surfaces, and sexes in most traits. There were some distinct differences among surfaces on both sexes for some movement traits. For example, metal consistently yielded the lowest average speed, acceleration and total distance in the control groups. Galvanization is a widely used industrial process that includes a thin layer of zinc applied to steel or iron to prevent material corrosion [66]. Exposure to different zinc chemical forms has been reported to disrupt insect physiology and behavior, impairing their ability to forage and locate mates [67,68]. Therefore, the contact of *T. confusum* with zinc coating could explain the differentiation of mobility on the metal surface. However, further research on the mobility effects of galvanized vs. non-galvanized steel on insects is required to evaluate this important finding. Another important observation was the low exploration mean values on glass, recorded in the control groups. Adults of *T. confusum* depend on their tarsal hook structures for adhesion and grip on surfaces, in order to effectively walk [44]. This reduction may be attributed to the smooth and homogeneous nature of glass, which can limit grip and increase slip events during locomotion in flour beetles [69]. Consequently, insects allocate more effort and energy to maintain their stability during locomotion, reducing their active exploratory behavior in the glass arena. The varied differences observed in certain mobility traits among surfaces in the control groups, such as average speed, average acceleration, or exploration rate, were eliminated after chlorfenapyr exposure. This trend was evident at LC_10_ and LC_30_ in most combinations, suggesting that chlorfenapyr can induce substantial locomotor impairment even at low concentrations. These results indicate that chlorfenapyr exposure was the main factor affecting the behavior of *T. confusum*, whereas surface type had a limited and statistically insignificant influence on the recorded responses. In contrast, mortality bioassay results have documented that surface texture and porosity often affect insecticidal efficacy by altering the availability of the a.i. on treated surfaces [70]. The current multi-trait analysis of the negative effects of chlorfenapyr on *T. confusum* behavior can aid in the collection of vital information on the biology of this pest. Reduced speed and acceleration indicate decreased responsiveness to stimuli, thus limiting the beetle’s ability to navigate properly or escape unsuitable conditions rapidly (e.g., temperature, RH) [71,72]. Additionally, mobility disruption characterized by reductions in exploration rate and covered distances, or increases in freezing time, is also correlated with low environmental spatial coverage, e.g., storage environments [73,74]. Within the genus *Tribolium*, mobility impairment has also been linked with negative effects on reproductive traits, including suitable mate detection, courtship, and copulation attempts or success [75,76,77].

The absence of significant sex-related differences among the vast majority of the assessed mobility endpoints across all tested surfaces and LC treatments likely reflects the weak size difference between *T. confusum* [77]. Recently, Kavallieratos et al. [78] supported that differences in size between sexes of *Trogoderma granarium* Everts (Coleoptera: Dermestidae) had a strong effect on the mobility of the adults. Females exhibited lower walking duration under chlorfenapyr exposure at LC_30_ than males (426 s and 502 s, respectively), an outcome that the authors attribute to the size difference, since females are 1.4 times larger than the males of this species [79].

Overall, our findings establish a strong base for the importance of automation in the behavioral evaluation of *T. confusum*. The recording and analysis of characteristics associated with motor function using ToxTrac allowed the critical assessment of toxicological effects of chlorfenapyr at low concentrations, which is impossible to capture with the naked eye. The fact that ToxTrac is freely available and the whole experimental setup can be arranged at low cost makes the entire procedure easily replicable and applicable to different protocols. This study highlights the differentiation of the motor activity of *T. confusum* adults when occurring on various surface materials. Future research should incorporate more experimentation steps and parameters, such as different pest species or exposure methods in varying environmental settings (food availability, temperature, RH) to further understand the effects of sublethal concentrations of insecticides on stored-product insect behavior. Testing also a wide spectrum of a.is., including green pesticides due to their promising insecticidal properties [80] will further benefit the research related to behavioral toxicology. Since behavioral studies on *T. confusum* are limited, the implementation of automated approaches can offer deeper knowledge of this species faster and thereby develop focused control strategies. Another important aspect deals with the evaluation of the intra- and inter-specific interactions of stored-product pests with the use of ToxTrac in order to unlock their behavioral responses.

## Figures and Tables

**Table 1:** Contact toxicity of chlorfenapyr on *Tribolium confusum* adults.

Surface	Concentration	LC_10_ (95% CI)	LC_30_ (95% CI)	LC_50_ (95% CI)	*χ*^2^, df, *p*
Cement	mg a.i./cm^2^	0.00838 (0.00595–0.0105)	0.0131 (0.0104–0.0152)	0.0178 (0.0153–0.0197)	107, 73, 0.006 *
Plastic	mg a.i./cm^2^	0.00475 (0.00270–0.00675)	0.00822 (0.00553–0.0105)	0.0120 (0.00906–0.0144)	62.8, 73, 0.80
Glass	mg a.i./cm^2^	0.00403 (0.00175–0.00633)	0.00688 (0.00375–0.00957)	0.00996 (0.00635–0.0128)	58.9, 73, 0.89
Metal	mg a.i./cm^2^	0.00463 (0.00221–0.00696)	0.00763 (0.00447–0.0103)	0.0108 (0.00727–0.0135)	59.9, 73, 0.86

LC_10_, LC_30_, and LC_50_, are lethal concentrations that kill 10%, 30%, and 50% of exposed beetles, respectively. 95% CI, lower and upper limits of the 95% confidence interval. The comparisons were conducted within each surface. * Significant (*p* ≤ 0.05).

**Table 2:** ANOVA parameters for main effects and associated interactions for the mobility traits of *Tribolium confusum* adults exposed to LC_10_ and LC_30_ of chlorfenapyr (error df = 719).

Mobility Traits		Average Speed (mm/s)		Average Acceleration (mm/s^2^)		Total Distance (m)		Exploration Rate (%)		Mobility Average Speed (mm/s)		Total Time Frozen (s)	
Source	df	*F*	*p*	*F*	*p*	*F*	*p*	*F*	*p*	*F*	*p*	*F*	*p*
Sex	1	0.1	0.33	1.3	0.26	0.9	0.36	0.1	0.71	0.5	0.48	7.6	0.01 *
Material	3	6.8	0.01 *	13.6	<0.01 *	10.0	<0.01 *	54.0	<0.01 *	3.2	0.02 *	18.1	<0.01 *
Treatment	2	173.2	<0.01 *	265.9	<0.01 *	140.9	<0.01 *	1.4	0.24	0.6	0.61	64.8	<0.01 *
Sex × material	3	0.9	0.42	2.2	0.08	1.3	0.27	156.6	<0.01 *	165.3	<0.01 *	1.8	0.15
Sex × treatment	2	1.0	0.38	1.2	0.30	1.0	0.39	2.4	0.10	1.0	0.36	2.0	0.14
Material × treatment	6	4.2	0.01 *	6.3	<0.01 *	5.6	<0.01 *	7.7	<0.01 *	5.4	<0.01 *	3.8	0.01 *
Sex × material × treatment	6	2.3	0.03 *	3.2	0.01 *	2.2	0.04 *	1.6	0.13	2.3	0.04 *	3.3	0.01 *

* Significant (*p* ≤ 0.05).

## Data Availability

Data is contained within the article.

## Supplementary Materials

The following supporting information can be downloaded at: https://www.mdpi.com/article/10.3390/insects17080801/s1, Table S1. Mean values ± SE of mobility traits of males and females of *Tribolium confusum* adults exposed to LC_10_ and LC_30_ of chlorfenapyr and controls on four surfaces.
