# Correction: Filintas et al. Assessing the Mobility Disruption of *Tribolium confusum* Under Sublethal Concentrations of Chlorfenapyr Through Video Tracking Software. *Insects* 2026, *17*, 801

**DOI:** 10.3390/insects17101079

**Published:** 2026-10-19

**Authors:** Constantin S. Filintas, Nickolas G. Kavallieratos, Maria C. Boukouvala

**Affiliations:** Laboratory of Agricultural Zoology and Entomology, Agricultural University of Athens, 75 Iera Odos str., 11855 Athens, Greece; kfilintas@aua.gr (C.S.F.); nick_kaval@aua.gr (N.G.K.)

Error in Supplementary Material (Table S1)

In the original publication [1], there was a mistake in the **Supplementary Material (Table S1)** regarding the mean values ± SE of the mobility traits of males and females of *Tribolium confusum* adults exposed to LC_10_ and LC_30_ of chlorfenapyr and the controls on four surfaces as published.

The numbers for cement were the same as those for plastic and there was a typo in total distance (glass/females/control). The corrected **Table S1** appears below. The authors state that the published paper is completely unaffected. This correction was approved by the Academic Editor. The original publication, concerning the Supplementary Material (Table S1) only, has been updated.

**Supplementary Materials:** The following supporting information can be downloaded at: https://www.mdpi.com/article/10.3390/insects17080801/s1, Table S1. Mean values ± SE of mobility traits of males and females of *Tribolium confusum* adults exposed to LC_10_ and LC_30_ of chlorfenapyr and controls on four surfaces.
