# Supplementary material for: Assessing the Mobility Disruption of Tribolium confusum Under Sublethal Concentrations of Chlorfenapyr Through Video Tracking Software
Source: Insects. 2026 Aug 3;17(8):801. doi: 10.3390/insects17080801 (PMC13513079; doi:10.3390/insects17080801)
Supplement: Supplementary file 1 [file insects-17-00801-s001.zip › insects-4428898-supplementary.pdf]

Table S1. Mean values ± SE of mobility traits of males and females of *Tribolium confusum* adults exposed to LC_{10} and LC_{30} of chlorfenapyr and controls on four surfaces.

Surface	Sex	Treatment	Average speed (mm/s)	Average acceleration (mm/s²)	Total distance (m)	Exploration rate (%)	Mobility average speed (mm/s)	Total time frozen (s)
Cement	Females	Control	63.8 ± 5.2	68.4 ± 4.7	20.6 ± 1.8	55.1 ± 3.6	63.7 ± 5.6	4.6 ± 1.6
		LC_{10}	37.7 ± 6.6	30.6 ± 2.6	10.7 ± 1.6	27.1 ± 3.9	32.1 ± 4.2	31.3 ± 11.1
		LC_{30}	33.8 ± 3.2	30.3 ± 2.2	11.3 ± 1.2	20.2 ± 2.0	37.5 ± 3.1	47.4 ± 10.6
	Males	Control	87.9 ± 10.3	89.8 ± 8.9	29.0 ± 0.4	58.2 ± 3.9	92.0 ± 10.1	15.8 ± 3.9
		LC_{10}	37.8 ± 9.1	27.5 ± 2.2	11.5 ± 1.1	28.8 ± 3.5	38.2 ± 9.6	20.51 ± 9.0
		LC_{30}	26.2 ± 2.6	24.1 ± 1.1	8.6 ± 1.0	18.1 ± 2.2	27.3 ± 2.5	21.5 ± 6.5
Glass	Females	Control	75.3 ± 4.2	63.9 ± 2.8	35.1 ± 10.0	26.6 ± 2.6	89.0 ± 10.2	10.5 ± 5.3
		LC_{10}	30.2 ± 2.0	26.7 ± 1.2	9.0 ± 0.9	14.3 ± 1.3	30.4 ± 2.9	22.9 ± 6.6
		LC_{30}	27.2 ± 1.8	23.6 ± 1.3	6.0 ± 0.7	10.3 ± 1.0	20.8 ± 2.2	36.1 ± 11.3
	Males	Control	77.2 ± 4.4	68.2 ± 3.6	29.0 ± 2.9	22.3 ± 3.1	95.8 ± 9.2	11.8 ± 5.5
		LC_{10}	30.3 ± 2.7	26.1 ± 1.2	8.2 ± 1.3	10.3 ± 1.5	28.9 ± 4.2	34.2 ± 10.8
		LC_{30}	24.8 ± 1.7	21.5 ± 1.2	8.7 ± 1.2	11.3 ± 1.2	28.1 ± 3.1	49.5 ± 7.9
Metal	Females	Control	52.3 ± 6.1	41.9 ± 3.9	15.7 ± 1.9	52.2 ± 2.7	54.8 ± 6.0	12.5 ± 3.7
		LC_{10}	26.4 ± 3.2	29.1 ± 3.2	7.2 ± 0.6	25.1 ± 2.8	30.0 ± 3.5	27.7 ± 5.3
		LC_{30}	29.0 ± 3.7	26.1 ± 3.0	8.9 ± 1.0	27.8 ± 3.8	32.2 ± 3.8	38.2 ± 8.0
	Males	Control	42.7 ± 2.6	39.6 ± 2.1	10.0 ± 0.9	40.0 ± 3.2	43.0 ± 2.6	22.0 ± 4.9
		LC_{10}	29.7 ± 3.4	25.7 ± 2.8	10.0 ± 1.4	33.0 ± 4.4	34.6 ± 3.3	49.8 ± 7.7
		LC_{30}	19.5 ± 3.3	18.3 ± 2.6	6.9 ± 1.4	24.8 ± 4.0	25.4 ± 3.4	83.7 ± 10.0
Plastic	Females	Control	98.6 ± 8.7	76.5 ± 5.8	31.2 ± 2.8	45.2 ± 3.0	103.2 ± 9.2	5.3 ± 2.8
		LC_{10}	27.6 ± 2.4	23.0 ± 1.6	8.8 ± 0.8	29.3 ± 3.4	29.1 ± 2.5	52.2 ± 9.5
		LC_{30}	26.0 ± 4.4	19.1 ± 5.2	8.2 ± 1.4	16.0 ± 2.1	32.9 ± 4.6	100.9 ± 15.5
	Males	Control	81.1 ± 8.6	58.0 ± 5.2	2.5 ± 2.8	48.0 ± 3.3	82.5 ± 8.8	16.4 ± 4.4
		LC_{10}	27.4 ± 2.7	28.7 ± 2.5	10.5 ± 1.4	36.7 ± 2.1	30.4 ± 2.7	27.2 ± 5.8
		LC_{30}	26.6 ± 4.5	20.4 ± 2.6	7.9 ± 1.3	14.9 ± 2.3	30.3 ± 4.7	79.3 ± 14.5
